# Physiological Synchrony Predict Task Performance and Negative Emotional State during a Three-Member Collaborative Task

**DOI:** 10.3390/s23042268

**Published:** 2023-02-17

**Authors:** Mohammed Algumaei, Imali Hettiarachchi, Rakesh Veerabhadrappa, Asim Bhatti

**Affiliations:** Institute for Intelligent Systems Research and Innovation, Deakin University, Waurn Ponds, VIC 3216, Australia

**Keywords:** team performance, physiological synchrony, emotional state, MdRQA

## Abstract

Evaluation of team performance in naturalistic contexts has gained popularity during the last two decades. Among other human factors, physiological synchrony has been adopted to investigate team performance and emotional state when engaged in collaborative team tasks. A variety of methods have been reported to quantify physiological synchrony with a varying degree of correlation with the collaborative team task performance and emotional state, reflected in the inconclusive nature of findings. Little is known about the effect of the choice of synchrony calculation methods and the level of analysis on these findings. In this research work, we investigate the relationship between outcomes of different methods to quantify physiological synchrony, emotional state, and team performance of three-member teams performing a collaborative team task. The proposed research work employs dyadic-level linear (cross-correlation) and team-level non-linear (multidimensional recurrence quantification analysis) synchrony calculation measures to quantify task performance and the emotional state of the team. Our investigation indicates that the physiological synchrony estimated using multidimensional recurrence quantification analysis revealed a significant negative relationship between the subjectively reported frustration levels and overall task performance. However, no relationship was found between cross-correlation-based physiological synchrony and task performance. The proposed research highlights that the method of choice for physiological synchrony calculation has direct impact on the derived relationship of team task performance and emotional states.

## 1. Introduction

Implementing unobtrusive measurements and a variety of sensor technologies to evaluate team performance in naturalistic contexts has gained popularity in recent decades [1]. Physiological synchrony (PS), referred to as the association of behavioural, physiological, or emotional activities over time between two or more individuals, has been adopted to investigate team performance and emotional state in the human factors literature [2,3,4]. Physiological patterns of the collaborative teams provide valuable insight about tasks, where inter-subject interactions can regulate the team performance outcomes [5] and synchronised physiological signals may indicate better performance [6].

In the last few decades, researchers have examined the relationship between PS and task performance. For example, Henning et al. [7] found that synchrony between dyads will increase with the task performance during a computer-based task. In another study [8], authors found that dyads who performed better in a building-clearing task were associated with higher PS. During a team task, it is expected that a higher level of PS would be associated with better performance when there are matching physical and cognitive demands of teammates. However, there are studies that have shown the opposite relationship using cooperative tasks in which team members are assigned to differentiated roles. For example, Strang and his colleagues [9] found that dyads’ performance is negatively associated with the PS during a cooperative task (Quadra game-play).

Many previous studies have investigated the relationship between PS and different team aspects, however, in dyadic contexts [6,7,10]. This has raised concerns among authors [11] as to whether interactions between pairs of people could be considered a team activity. In this light, our investigation of studies employing three or more members in a team fails to demonstrate the true and consistent effects of PS on task performance [12,13,14,15,16,17]. For example, Dindar and colleagues [12] examined the relationship between PS and task performance for three-member teams working on a computer-based simulation task while their electrodermal activity (EDA) was recorded. Similarly, authors in [13] investigated the PS in a three-member team performing a competitive origami boat-building task. These studies, however, have failed to detect true and consistent effects of PS on task performance.

In the literature, several methods were used to quantify the PS in teams of three or more members using different analysis levels. The dyadic-level was employed using cross-correlation (CC) [6,15] and cross-recurrence quantification analysis (CRQA) [13,17] between dyads within a team. Authors in [12] employed a team-level analysis using multidimensional recurrence quantification analysis (MdRQA). The current literature demonstrates inconclusive in findings about the way in which PS is reflected in task performance, and little is known about the effect of the chosen analysis and level of the analysis used. Further studies are therefore required to determine the relationship between PS and task performance in three or more member teams, as an alternative to measuring a group’s PS using the collective representation of team member’s dyadic synchrony. [14]. Such studies might enable newer objective measures for evaluating team performance and help to design effective collaborative scenarios that facilitate team performance.

In this light, we designed a study engaging three members in a collaborative computer-based team task (the Tank Battle task will be presented in Section Tank Battle Team Task). The MdRQA and CC methods were employed to calculate the PS between three team members. We further investigated the effect of these methods as a measure of synchrony and its relationship with task performance. We extended the investigation by exploring the relationship between the synchrony measure and team-level aggregation of self-reported NASA TLX. The frustration level item in the NASA-TLX scale is used to evaluate the emotional state of the team in terms of their level of frustration [18]. By measuring this level, we can gain a better understanding of the overall emotional state of the team.

This will be achieved via the following research questions:

RQ1: Is the relationship between the team’s PS and task performance affected by the method used for synchrony calculation?

RQ2: Further, is the association between the PS and the subjective feedback affected by the PS calculation method?

To address the research questions, we employed regression models to objectively predict task performance and self-reported NASA-TLX scores using the PS measures.

**Outline** The rest of this paper is organised as follows: The first section “Materials and methods”, describes the experimental task and details the subjects and procedure. It also discusses the data recording and preparation. The second section introduces the measures used in this study to evaluate the team performance. After that, the section “Results”, shows the findings of the current study, and discusses these findings in the section “Discussion”. Finally, we discuss the limitations and future direction of the results in our study.

## 2. Materials and Methods

### 2.1. Tank Battle Team Task

**Figure 1:**
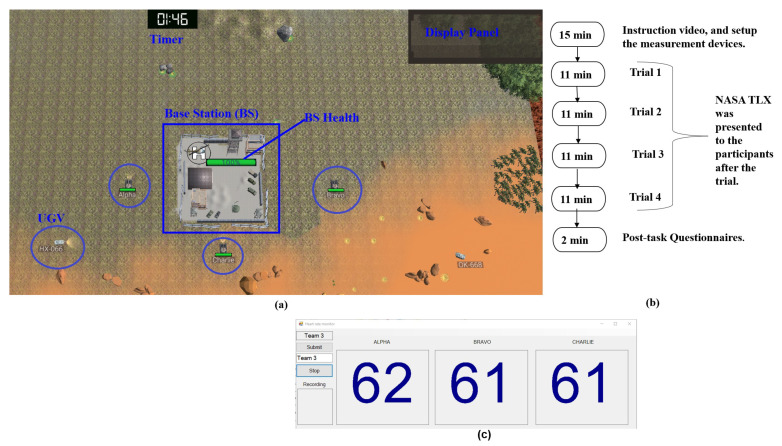
Experimental setup overview and timeline of the experiment. (**a**) Operating window. (**b**) The estimated timeline of the experiment. (**c**) Custom data app developed to interface the three Polar H10 sensors, the values represent the instantaneous heart rate in beats per minute for each of the participants.

In this study, we used a collaborative computer-based simulation task, referred to as Tank Battle (TB) [15,19,20]. The main operation window as shown in Figure 1a contained three components: a display panel that conveys critical information to each member, a timer window, and base station (BS) which has the BS health indicator. The task involved three members referred to as Alpha, Bravo, and Charlie for communication purposes, and a manager in charge of starting the simulation task.

The main aim of the task is to protect the BS and maintain the health as high as possible. There are two different variants of unknown ground vehicles (UGV) approaching the BS. The enemy variant attacks the BS by firing projectiles, which reduces the health and the friend variant provides aid to the BS which increases the health. In order to achieve the task’s aim, the team members have to communicate with other members to identify the type of incoming variants and work together strategically, collaboratively, and cooperatively to protect the BS from the enemy variant attacks, and avoid destroying the friend variants so that the BS successfully receives the aid. Each team member is assigned a semi-autonomous battle tank (BT) that can be controlled by a computer’s mouse. Members can navigate their BT’s to a location on the map and click on a UGV to attack and destroy.

To encourage effective collaboration through communication, two identification numbers were assigned to each UGV. A global identification (GID) is used to reference the UGV and it is visible to all three members, and a unique identification number (UID) was unique for each member. The UID is a number between 0 and 25 in multiples of 5, and the sum of the three UIDs decides whether the incoming UGV is a friend or an enemy. Each member can view the UID by placing the mouse over a UGV of interest and share the number with other members of the team. If the sum of the three UIDs observed by the team is equal to 30, then the UGV is identified as an enemy and the team needs to act and destroy it, else it is a friend and no further action is required. Hence, effective communication is key for successful task performance.

### 2.2. Participants

In this study, 17 teams of 3 members each participated, However, data from 3 teams were not included in the analysis due to missing or unreliable data from one or more team members. The average age of the 42 participants was 22.6 years for males and 22.4 years for females, with a range of 18 to 32 years. The data were collected during a one-hour period in the morning. Participants completed four trials of a simulated task. To ensure accurate results, participants were instructed to avoid vigorous exercise and alcohol in the 24 h prior to the experiment, as well as coffee, tea, or other stimulating beverages in the 2 h prior to the experiment.

### 2.3. Procedure

The study received approval as low-risk research from the Human Research Ethics Advisory Group of the Faculty of Science and Technology at Deakin University in Australia. After participants signed the written informed consent, they wore the Polar H10 chest strap on their upper rib cage (following the H10 wearing guidelines [21]). Triads were randomly seated in front of the computers with new identifications assigned as Alpha, Bravo, and Charlie. Subsequently, participants watched a video explaining the rules and objectives of the task. Following the instruction video, participants completed a 1 min practice trial. These procedures were aimed at getting the teams familiarised with the task. The experimental task consisted of four trials with each trial lasting 11 min; Figure 1b shows the estimated time of the total experiment. Participants completed NASA-TLX questionnaires at the end of each trial and the post-experiment cooperation questionnaires at the end of the simulated task [18].

### 2.4. Physiological Data

Polar H10 sensors were used to collect the cardiac inter-beat-intervals (IBI) activity for each participant [21]. The IBI data contain the time elapsed between two successive R-waves of the QRS complex (RR interval). The three sensors use surface electrodes enabling a non-invasive method to acquire the data and store them in the server via Bluetooth in comma-separated value (.csv) file format. A custom Windows application was developed to start and end the recording. Figure 1c shows the application interfacing the three Polar sensors where the values in the figure represent the instantaneous heart rate (HR) in beats per minute for each of the team members.

#### Data Preparation

The IBI data for the study were prepared and analysed using MATLAB software (version R2020a, MathWorks). The IBI time series were cleaned and pre-processed for each team-trial member. The start and end times of the trials were used to synchronise the IBI series data for each team member and trial combination. Then, a time axis starting with zero was derived for the IBI time series data. The IBI series were then interpolated to four times the number of samples and any unwanted data exceeding 1000 ms duration were removed. A quotient filter was applied during the data pre-processing to eliminate artifacts and non-natural beats [22,23].

Following the cleaning and resampling process, a moving-window-based approach was employed to generate the HRV data. The HRV data weere calculated for a 1 min window with (1/3) min overlapping. This results in a time series for each team member during four trials. The 1 min window was chosen as the shortest reliable window for HRV measures [6,24]. For each window, different statistical time-domain HRV measures are derived, which capture the variation in IBI, such as the mean of RR intervals (mRR), the root mean square of the differences of successive RR intervals (rMSSD), and the standard deviation of RR intervals (SDNN) [25,26]. This resulted in a time series for mRR, rMSSD, and SDNN with x values for each team–trial–participant combination. These HRV parameters time series were used to generate team PS as described below.

## 3. Measures of Team Performance

### 3.1. Subjective Measures

Subjective measures of workload during the task were collected via questionnaires in the form of feedback from participants. Researchers consider these techniques the most direct measures of team members workload as they are flexible and no special equipment for data collection is needed [27]. NASA-TLX is one of the most widely used to evaluate overall subjective workload and is used in this study to obtain participant feedback on the team task [18]. NASA-TLX analyses three demands of requirements that concern individuals: physical, temporal, and mental. It also analyses three items related to the willingness of individuals: performance, effort, and frustration.

#### NASA Task Load Index

After each trial, the NASA-TLX questionnaire was presented to each team member, where they provided feedback on perceived levels of physical demand, mental demand, temporal demand, performance, effort, and frustration. Participants responded to NASA-TLX questions on a scale of 0–10 such that scores of 0 and 10 represented the minimum and the maximum load index, respectively.

The NASA-TLX scores for all team–trial–participant combination for the Tank Battle Task is as follows,

- Physical Demand (PD), M = 2.49, SD = 1.28, (0: not demanded, 10: demanded)
- Temporal Demand (TD), M = 4.65, SD = 1.11, (0: not demanded, 10: demanded)
- Performance (Perf), M = 5.55, SD = 1.28, (0: bad performance, 10: good performance)
- Effort (Eff), M = 5.22, SD = 1.18, (0: no effort needed, 10: effort needed)
- Frustration (Frust), M = 3.69, SD = 1.47, (0: not frustrated, 10: frustrated)
- Mental Demand (MD), M = 5.46, SD = 1.19, (0: not demanded, 10: demanded)

**Figure 2:**
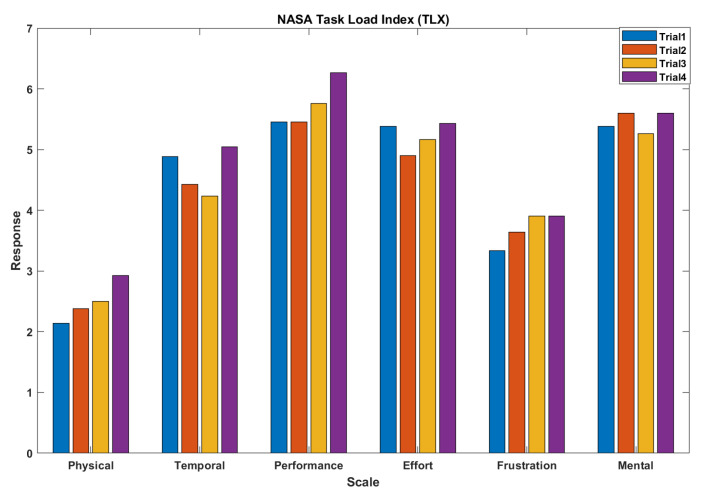
Average task load index experienced by participants in trial episodes.

Figure 2 shows the average load indices experienced by participants in each trial. From the results, it could be understood that the overall physical demand required to perform the task was relatively less, as some people are physically strong whereas others could be a little weak. This could induce bias in the performance. Thus, we intend to exclude it when we apply multiple regression to investigate the relationship between the synchrony and the load index response.

### 3.2. Physiological Synchrony Calculation

#### 3.2.1. Cross Correlation (CC)

For each HRV parameter, physiological synchrony (PS) was measured by pair-wise CC within the team dyads (Alpha-Bravo, Bravo-Charlie, and Alpha-Charlie). Team synchrony was then calculated by taking the average of three-pair values.

#### 3.2.2. Multidimensional Recurrence Quantification Analysis (MdRQA)

MdRQA is a powerful technique for analysing time series data. It is a generalisation of traditional recurrence analysis, which is used to study the dynamics of a single time series. MdRQA allows for the analysis of multiple time series simultaneously by measuring the recurrence of patterns in a multidimensional phase space [28]. It was developed to characterise the behaviours of time series data that provide multiple interdependent variables, potentially exhibiting non-linear behaviours over time [29]. The choice of the MdRQA over the other recurrence quantification analysis techniques is based on the need to assess the synchrony among three signals from three team members simultaneously, which is not applicable in recurrence quantification analysis (RQA) or CRQA. The use of MdRQA in this study is to capture the effect of the team-level dynamics by quantifying the synchrony of three signals unlike the dyadic-level measure based on CC. Several MdRQA measures were developed to quantify the team synchrony by examining the team as a dynamical system [14]. These measures are interrelated and each captures a different aspect of the dynamic system. Hence, four measures were adopted to conduct this analysis.

- Recurrence rate (REC)—a key metric in MdRQA that is used to measure the degree of recurrence in a time series. It is defined as the proportion of recurrent points in the phase space. A high recurrence rate indicates that many points in the phase space are recurrent, while a low recurrence rate indicates that few points are recurrent [28].
- Determinism (DET)—used to measure the degree of predictability in a time series. It is defined as the proportion of recurrent points that form diagonal lines in the recurrence plot. A high determinism indicates that the recurrent points form many diagonal lines, which suggests that the time series is highly predictable, while a low determinism indicates that the recurrent points form few diagonal lines, which suggests that the time series is less predictable [29,30].
- Average diagonal line (ADL)—used to measure the average length of diagonal lines in the recurrence plot. It is defined as the average number of points on a diagonal line in a recurrence plot. A high ADL indicates that the recurrent points form long diagonal lines, which suggests that the time series is highly predictable, while a low ADL indicates that the recurrent points form short diagonal lines, which suggests that the time series is less predictable [28,31].
- Maximum diagonal line (MDL)—used to measure the maximum length of diagonal lines in the recurrence plot. It is defined as the highest number of points on a diagonal line in a recurrence plot. A high MDL indicates that the recurrent points form long diagonal lines, which suggests that the time series is highly predictable, while a low MDL indicates that the recurrent points form short diagonal lines, which suggests that the time series is less predictable [28,30].

### 3.3. MdRQA Parameter Estimation

The recurrence analysis was conducted by projecting the time series into a phase space using the method of time-delayed embedding. The time series were plotted against themselves with a time lag, as determined by the delay parameter (DEL), and the number of times the data was plotted against itself was determined by the dimension parameter (DIM). To reduce the influence of the magnitude of the signal, the time series were normalised before the embedding procedure. For the HRV data, the data for each individual in the team was normalised using a z-score before analysis to minimise the effects of the magnitude of the signal on the estimation of synchrony. The HRV parameters for the three members of each team were then embedded into the phase space to calculate the recurrence plot measures. The embedded parameters were determined using the average mutual information (AMI) function to estimate the delay parameter and the false nearest neighbor (FNN) function to estimate the dimensionality (DIM) of the phase space as described in reference [32].

Using a univariate approach, for each time series (i.e; N*team X 3*members) the AMI function was employed to estimate the DEL parameter for each individual data set. Then, the first local minimum of the AMI function for each data set was identified and the round up values were averaged. These rounded values were used for all the data set in MdRQA. The FNN function also was employed for each data set to estimate the DIM parameter. The first local minimum of the function was chosen for each data set, averaged across all sets, with the value then divided by three and rounded up and used for all data sets in MdRQA. Using a multivariate approach, similar procedures for each team’s data sets and following the same steps for estimating the DEL and DIM parameters were employed.

**Figure 3:**
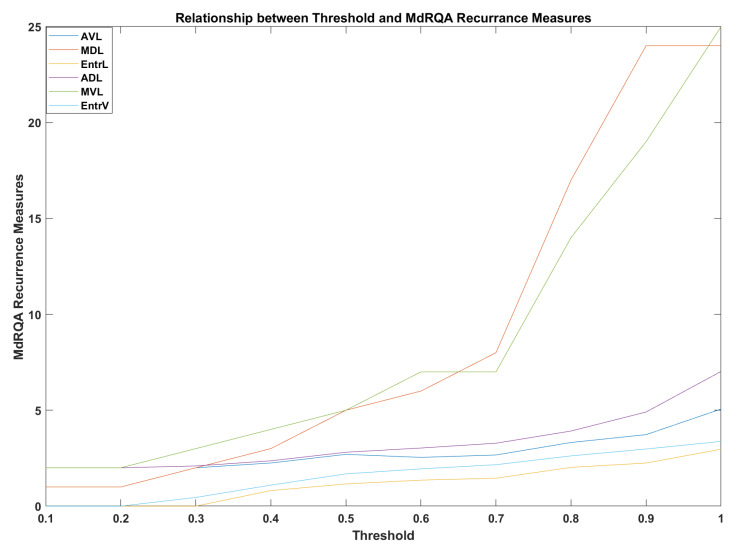
Relationship between threshold and MdRQA recurrence measures for mRR.

The calculated values for DEL = 2 and DIM = 1 using mRR parameter and the Euclidean norm were used to rescale the phase space along with a threshold of 0.7. The threshold was calculated from 0.1 to 1 and from Figure 3 below we can see the change as increasing the threshold the MdRQA measures will increase. However, at 0.7 an intense change was noticed; hence, it has been chosen to be used in the analysis. Similar procedures were followed for SDNN and rMSSD which give similar values.

## 4. Statistical Analysis

To quantify the relationship between the PS measures and the self-reported NASA-TLX, we used a multiple regression model. Since MdRQA and CC measures were employed as a measure of synchrony between team members, and by using teams’ NASA TLX as an independent variable, the multiple linear regression model could be written as follows:

(1) $$Y_{i}=\;\beta_{0}+\beta_{1}TD+\beta_{2}Perf+\beta_{3}Eff+\beta_{4}Frust+\beta_{5}MD$$

where *Y* denotes a PS measures; REC, DET, ADL, MDL, and CC, and *i* denote the HRV parameter, and TD, Perf, Eff, Frust, and MD are distinct independent variables, $\beta_{0}$ is the value of the dependent variables when all of the independent variables are equal to zero, and $\beta_{1}$ through $\beta_{5}$ are the estimated regression coefficients.

## 5. Results

**Figure 4:**
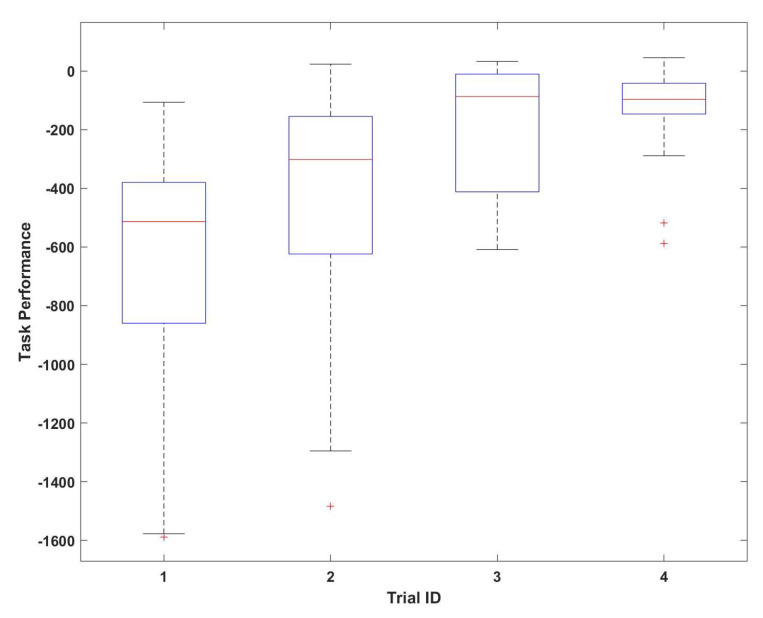
Variation of task performance throughout the trials.

One-way analysis of variance (ANOVA) was employed to observe if there was any statistical difference between the performance of several teams through trials. The results in Figure 4 indicate that the task performance has shown a significant increase (F(3,52) = 6.86 *p* < 0.0006) throughout the trials. These findings revealed that the experience and familiarity with the task will improve the task performance.

### 5.1. Relation between Synchrony and Task Performance

**Figure 5:**
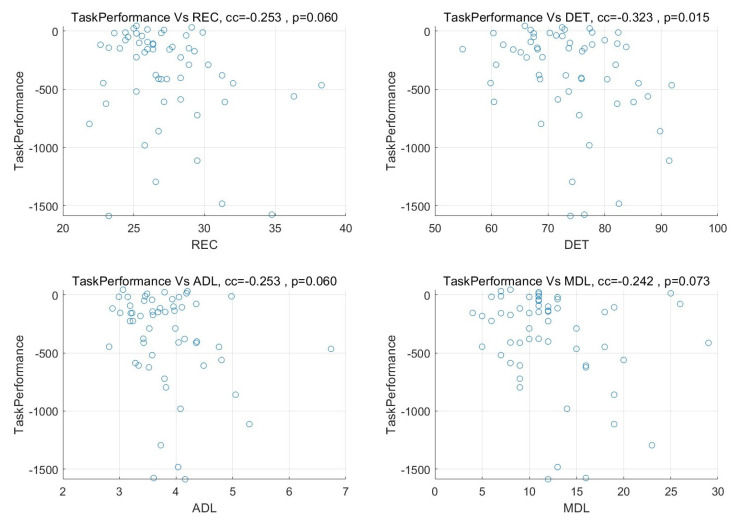
The scatter plot for MdRQA measures and task performance for mRR.

A Pearson correlation analysis using SPSS software was implemented to see the relationship between task performance and physiological synchrony calculated using MdRQA and CC measures among the three team members. In the Table 1, MdRQA recurrence measures such as DET and MDL were significantly correlated with task performance using mRR and SDNN features, while the rMSSD feature did not show any significant correlation with task performance (see the scatter plot in Figure 5 for MdRQA measures using mRR feature).

Proceeding from the correlation analysis, to further assess whether PS measures can be used as an objective tool to predict task performance, a simple linear regression analysis was conducted. The dependent variable in this analysis is task performance and the independent variables are the non-linear measures (i.e., REC, DET, MDL, and ADL) and linear measures (pair-wise CC ).

From the results in Table 2, the maximum diagonal line for SDNN feature demonstrated significant results relative to other MdRQA measures with adj*R*^2^ of 0.2 with *p*-value < 0.01. On the contrary, the average pair-wise CC within the team showed no significant results for the three features of HRV. Overall, the PS measured using the MdRQA was observed to be significant in some variables, such as MDL and DET, while the CC analysis shows no significant using different HRV parameters. The results show that the relationship between a team’s PS and task performance would be affected by the method used to calculate the synchrony (RQ1). It clearly shows that for the current task, the MdRQA measures of PS were sensitive and captured different information about the synchrony dynamics of a time series.

### 5.2. Relation between Synchrony and Subjective Measures

To address the second research question (RQ2), a multiple linear regression model was employed to quantify the relationship between subjective feedback and the synchrony measured by MdRQA and CC. Table 3 includes the results of multiple linear regression between MdRQA (REC) and CC measures with subjectively reported NASA-TLX scores, while other MdRQA measures can be seen in Appendix A.

From the Table 3, we can see that the synchrony measured by MdRQA (REC) shows a significant negative relationship with the frustration level reported by team members using mRR and a positive relationship with temporal demand using SDNN. The mRR and SDNN parameters both demonstrated significant reported performance items, while the rMSSD parameter did not show any significant results on other NASA-TLX items. The synchrony measured by CC shows a significant negative relationship with the frustration level using mRR and rMSSD parameters. Both mRR and SDNN parameters show a significant positive relationship with temporal demand reported by team members.

The findings indicate that the method used to calculate synchrony did not impact the correlation between a team’s PS and subjective feedback (RQ2). In fact, both MdRQA (REC) and CC measures had a significant impact on subjective feedback, particularly in regards to the level of frustration reported by team members.

## 6. Discussion

The objective of this study was to examine the correlation between the physiological synchrony (PS) of three team members, as measured by inter-beat interval (IBI) data and task performance and subjective feedback during a team task. The linear measure of synchrony was calculated using time-domain HRV by taking the mean of the three pairwise cross-correlation values. Non-linear measures of HRV synchrony among the three team members were also evaluated using the MdRQA method.

Pearson correlation analysis was performed to investigate the relationship between PS and task performance. The findings in Table 1 showed significant effects of the synchrony variations between the MdRQA measures. The measures that are dependent on the distribution of diagonal lines of recurrent points were observed to offer highly sensitive measures. There was no impact on task performance using CC between team members using HRV features.

In regard to the first research question (RQ1), a simple linear regression model was employed to examine the ability of the PS calculated using CC and MdRQA measures to predict task performance. The regression model results were reported in Table 2. From the Table 2, MdRQA measures such as DET and MDL showed significant results compared to the CC measure, which can be considered more reliable and robust for measuring the PS for teams with more than two members. Our study supports the findings in another study [17] which emphasises the fact that synchronous dynamics in teams are not always well-captured in terms of dyadic interaction (using pairwise CC), but that such dynamics can be established using group-level representations.

In addressing the second research question (RQ2), a multiple linear regression model was used to analyse the relationship between PS and teams’ reported scores on NASA-TLX items. The results in Table 3 showed a negative relationship between synchrony and the frustration items of NASA-TLX using MdRQA (REC) and CC measures. The lack of or a non-significant relationship with other items can be attributed to the impact of NASA-TLX reporting and the subjectivity of individual experiences. Participants self-reported their experiences immediately after each trial, and it is possible that they based their responses on the valence of feedback (i.e., positive or negative) rather than their actual task experiences before the feedback [33,34].

Our study aimed to encourage team members to coordinate and cooperate to protect and maintain the good performance of the BS during a series of UGV attacks. Effective communication was critical for exchanging information about UGV variants and identifying friend or enemy units. Interpersonal coordination was essential for success in the task. However, the strategies developed by team members to improve performance led to conflicting results in terms of physiological synchrony. Our study supports previous research that found that PS between dyads in joint construction tasks decreases as task performance improves [35].

## 7. Limitation and Future Work

Authors in [12] found that the PS is not related to team task performance. They explained the non-significant relationship as effective collaboration depends on both synchronicity and complementarity of the interactions within a team. Furthermore, the type of task may influence how task performance and physiological synchronisation relate to one another in a specific data modality. Our study found an effect of PS on subjective and objective measures using MdRQA analysis for three team members during a team task. However, these results cannot be generalised to all teams as the relationship between synchrony and task performance may be influenced by various team attributes, developed strategies, and the differentiation of experimental task roles [9].

The study used only one physiological measure (ECG) to examine PS. Using multiple measures (such as EEG, eye gaze, and EDA) would offer a more comprehensive understanding of team dynamics and how subjective feedback and task performance relate to PS [1]. The study also only looked at teams of strangers, so it is uncertain if the findings apply to teams who are more familiar with each other, which is often the case in real-world team work. Therefore, future research should test the results with different experimental tasks and teams that vary in familiarity and task difficulty, and use multiple physiological measures.

## 8. Conclusions

The current study investigates the effects of various methods for calculating physiological synchrony among three team members on task performance and subjective feedback during a collaborative task. The findings indicate that non-linear MdRQA measures are more effective than linear CC measures in predicting overall team performance and capturing the dynamics between team members during a collaborative task. Our results reveal a significant relationship between PS calculated using MdRQA and task performance. However, further research is necessary to fully understand the impact of synchrony on task performance in collaborative settings.

## Figures and Tables

**Table 1:** Correlations between synchrony measures and task performance.

	mRR	SDNN	rMSSD
Measures	Task Performance	Task Performance	Task Performance
REC	−0.253 *	−0.195	0.004
DET	−0.323 **	−0.324 **	−0.213
ADL	−0.253 *	−0.119	−0.090
MDL	−0.242 *	−0.413 **	−0.113
CC	−0.075	−0.095	0.081

** Correlation is significant at the 0.05 level, * Correlation is significant at the 0.1 level.

**Table 2:** Linear regression model of task performance and MdRQA indicators. Column headers, from left: HRV features; MdRQA indicators and CC analysis; adj. $R^{2}$ adjusted R-squared values; model estimate; standard error of the model; t statistics; *p* significant value.

HRV Feature	Fitted	adj*R*^2^	Est	Std. Err	tStat	*p*-Value
	REC	0.05	−32.14	16.73	−1.92	0.06 *
	DET	0.09	−15.98	6.36	−2.51	0.01 **
mRR	ADL	0.05	−153.32	79.93	−1.92	0.06 *
	MDL	0.04	−18.63	10.17	−1.83	0.07 *
	CC	−0.01	−159.5	290.03	−0.55	0.58
	REC	0.15	−23.55	16.11	−1.46	0.14
	DET	0.09	−13.38	5.31	−2.52	0.01 **
SDNN	ADL	−0.004	−33.38	37.83	−0.88	0.38
	MDL	0.2	−40.89	12.26	−3.33	0.001 **
	CC	−0.009	−186.87	265.7	−0.70	0.48
	REC	−0.02	0.462	15.10	0.03	0.97
	DET	0.03	−10.76	6.72	−1.60	0.11
rMSSD	ADL	−0.01	−29.78	45.08	−0.66	0.51
	MDL	−0.006	−9.95	11.96	−0.83	0.40
	CC	−0.01	198.86	333.21	0.59	0.55

** Significance level indicated as *p* < 0.05, * Significance level indicated as *p* < 0.1.

**Table 3:** Multiple linear regression model of MdRQA and NASA-TLX scores. Column headers, from left: fitted NASA-TLX scores; model estimate; standard error of the model; t statistics; *p* significant value.

HRV Features	Fitted	Est	Std. Err	tStat	*p*-Value
REC_*mRR*_	Intercept	28.57	2.47	11.5	0.00
	TD	0.66	0.60	1.10	0.27
	Perf	−0.71	0.34	−2.07	0.04 **
	Eff	0.64	0.60	1.07	0.28
	Frust	−0.57	0.29	−1.95	0.05 *
	MD	−0.30	0.48	−0.63	0.53
REC_*SDNN*_	Intercept	31.66	2.64	11.97	0.00
	TD	1.20	0.64	1.86	0.06 *
	Perf	−0.82	0.369	−2.22	0.03 **
	Eff	−1.04	0.64	−1.61	0.11
	Frust	−0.04	0.31	−0.14	0.88
	MD	−0.04	0.52	−0.07	0.94
REC_*rMSSD*_	Intercept	24.6	3.11	7.90	0.00
	TD	0.60	0.75	0.79	0.42
	Perf	0.034	0.435	0.078	0.93
	Eff	−0.64	0.75	−0.85	0.39
	Frust	0.18	0.369	0.497	0.62
	MD	0.25	0.612	0.41	0.67
CC_*mRR*_	Intercept	0.09	0.15	0.62	0.53
	TD	0.06	0.036	1.78	0.08 *
	Perf	0.01	0.02	0.84	0.4
	Eff	−0.03	0.03	−0.82	0.41
	Frust	−0.04	0.017	−2.36	0.02 **
	MD	−0.02	0.029	−0.7	0.48
CC_*SDNN*_	Intercept	0.19	0.17	1.11	0.27
	TD	0.07	0.04	1.9	0.06 *
	Perf	−0.02	0.023	−0.84	0.4
	Eff	−0.03	0.04	−0.85	0.39
	Frust	−0.01	0.02	−0.66	0.5
	MD	−0.01	0.03	−0.55	0.58
CC_*rMSSD*_	Intercept	0.016	0.13	0.12	0.90
	TD	0.01	0.032	0.43	0.66
	Perf	−0.0007	0.01	−0.04	0.96
	Eff	0.02	0.03	0.91	0.36
	Frust	−0.03	0.01	−2.21	0.03 **
	MD	−0.01	0.02	−0.55	0.58

** Significance level indicated as *p* < 0.05, * Significance level indicated as *p* < 0.1.

## Data Availability

The data analysed for this study are available on reasonable request.

## Appendix A

**Table A1:** Multiple linear regression model of MdRQA and NASA-TLX scores. Column headers, from left: fitted NASA-TLX scores; model estimate; standard error of the model; t statistics; *p* significant value.

HRV Features	Fitted	Est	Std. Err	tStat	*p*-Value
DET_*mRR*_	Intercept	76.6	6.84	11.19	0.00
	TD	1.38	1.66	0.83	0.41
	Perf	−0.73	0.957	−0.76	0.45
	Eff	−2.27	1.66	−1.36	00.17
	Frust	−0.35	0.811	−0.43	0.66
	MD	1.44	1.34	1.07	0.28
ADL_*mRR*_	Intercept	4.20	0.55	7.57	0.00
	TD	0.043	0.13	0.31	0.75
	Perf	−0.07	0.07	−0.84	0.40
	Eff	−0.06	0.13	−0.48	0.63
	Frust	−0.09	0.06	−1.4	0.16
	MD	0.08	0.11	0.79	0.42
MDL*_mRR_*	Intercept	13.65	4.44	3.07	0.00
	TD	0.86	1.08	0.80	0.43
	Perf	−0.19	0.62	−0.31	0.75
	Eff	−1.32	1.08	−1.23	0.22
	Frust	−0.26	0.53	−0.49	0.62
	MD	0.67	0.87	0.76	0.45
DET*_SDNN_*	Intercept	77.8	8.11	9.59	0.00
	TD	0.9	1.97	0.46	0.65
	Perf	−1.75	1.13	−1.54	0.13
	Eff	−1.99	1.97	−1.01	0.31
	Frust	0.27	0.96	0.28	0.77
	MD	0.33	1.59	0.20	0.83
ADL*_SDNN_*	Intercept	4.55	1.23	3.69	0.00
	TD	0.16	0.30	0.53	0.59
	Perf	−0.16	0.17	−0.953	0.34
	Eff	−0.13	0.29	−0.45	0.64
	Frust	−0.01	0.14	−0.07	0.94
	MD	−0.009	0.24	−0.03	0.97
MDL*_SDNN_*	Intercept	13.007	3.26	3.98	0.00
	TD	0.31	0.79	0.39	0.69
	Perf	−0.44	0.45	−0.97	0.33
	Eff	−1.79	0.79	−2.25	0.03 **
	Frust	0.23	0.38	0.61	0.54
	MD	0.94	0.64	1.47	0.14
DET*_rMSSD_*	Intercept	69.03	6.63	10.4	0.00
	TD	2.07	1.61	1.28	0.21
	Perf	−1.02	0.92	−1.11	0.27
	Eff	−1.33	1.61	−0.82	0.41
	Frust	−0.54	0.78	−0.69	0.49
	MD	0.88	1.30	0.68	0.49
ADL*_rMSSD_*	Intercept	3.84	1.042	3.68	0.00
	TD	0.17	0.25	0.69	0.48
	Perf	−0.07	0.15	−0.54	0.58
	Eff	−0.09	0.25	−0.37	0.71
	Frust	−0.04	0.123	−0.35	0.72
	MD	0.01	0.20	0.05	0.96
MDL*_rMSSD_*	Intercept	9.71	3.71	2.61	0.01
	TD	−0.13	0.91	−0.15	0.87
	Perf	−0.37	0.52	−0.72	0.47
	Eff	−1.11	0.90	−1.23	0.22
	Frust	−0.26	0.44	−0.59	0.55
	MD	1.70	0.73	2.33	0.02 **

** Significance level indicated as *p* < 0.05, * Significance level indicated as *p* < 0.1.

## References

[1] Kazi S., Khaleghzadegan S., Dinh J.V., Shelhamer M.J., Sapirstein A., Goeddel L.A., Chime N.O., Salas E., Rosen M.A. (2021). Team physiological dynamics: A critical review. Hum. Factors.

[2] Ekman I., Chanel G., Järvelä S., Kivikangas J.M., Salminen M., Ravaja N. (2012). Social interaction in games: Measuring physiological linkage and social presence. Simul. Gaming.

[3] Järvelä S., Kivikangas J.M., Kätsyri J., Ravaja N. (2014). Physiological linkage of dyadic gaming experience. Simul. Gaming.

[4] Palumbo R.V., Marraccini M.E., Weyandt L.L., Wilder-Smith O., McGee H.A., Liu S., Goodwin M.S. (2017). Interpersonal autonomic physiology: A systematic review of the literature. Personal. Soc. Psychol. Rev..

[5] Ahonen L., Cowley B.U., Hellas A., Puolamäki K. (2018). Biosignals reflect pair-dynamics in collaborative work: EDA and ECG study of pair-programming in a classroom environment. Sci. Rep..

[6] Ahonen L., Cowley B., Torniainen J., Ukkonen A., Vihavainen A., Puolamäki K. (2016). Cognitive collaboration found in cardiac physiology: Study in classroom environment. PLoS ONE.

[7] Henning R.A., Boucsein W., Gil M.C. (2001). Social–physiological compliance as a determinant of team performance. Int. J. Psychophysiol..

[8] Elkins A.N., Muth E.R., Hoover A.W., Walker A.D., Carpenter T.L., Switzer F.S. (2009). Physiological compliance and team performance. Appl. Ergon..

[9] Strang A.J., Funke G.J., Russell S.M., Dukes A.W., Middendorf M.S. (2014). Physio-behavioral coupling in a cooperative team task: Contributors and relations. J. Exp. Psychol. Hum. Percept. Perform..

[10] Veerabhadrappa R., Hettiarachchi I.T., Bhatti A. Using Recurrence Quantification Analysis to Quantify the Physiological Synchrony in Dyadic ECG Data. Proceedings of the 2021 IEEE International Systems Conference (SysCon).

[11] Moreland R.L. (2010). Are dyads really groups?. Small Group Res..

[12] Dindar M., Järvelä S., Haataja E. (2020). What does physiological synchrony reveal about metacognitive experiences and group performance?. Br. J. Educ. Technol..

[13] Mønster D., Håkonsson D.D., Eskildsen J.K., Wallot S. (2016). Physiological evidence of interpersonal dynamics in a cooperative production task. Physiol. Behav..

[14] Baranowski-Pinto G., Profeta V., Newson M., Whitehouse H., Xygalatas D. (2022). Being in a crowd bonds people via physiological synchrony. Sci. Rep..

[15] Algumaei M., Hettiarachchi I., Veerabhadrappa R., Bhatti A. Physiological Compliance during a Three Member Collaborative Computer Task. Proceedings of the 2022 IEEE International Conference on Systems, Man, and Cybernetics (SMC).

[16] Fusaroli R., Bjørndahl J.S., Roepstorff A., Tylén K. (2016). A heart for interaction: Shared physiological dynamics and behavioral coordination in a collective, creative construction task. J. Exp. Psychol. Hum. Percept. Perform..

[17] Gordon I., Wallot S., Berson Y. (2021). Group-level physiological synchrony and individual-level anxiety predict positive affective behaviors during a group decision-making task. Psychophysiology.

[18] Hart S.G., Staveland L.E. (1988). Development of NASA-TLX (Task Load Index): Results of empirical and theoretical research. Advances in Psychology.

[19] Veerabhadrappa R., Hettiarachchi I.T., Bhatti A. Gaze Convergence Based Collaborative Performance Prediction in a 3-Member Joint Activity Setting. Proceedings of the 2022 IEEE International Systems Conference (SysCon).

[20] Veerabhadrappa R., Hettiarachchi I.T., Bhatti A. Using Eye-tracking To Investigate The Effect of Gaze Co-occurrence and Distribution on Collaborative Performance. Proceedings of the 2022 IEEE International Systems Conference (SysCon).

[21] Polar H10 Heart Rate Monitor + Chest Strap—Black. https://www.polar.com/au-en/sensors/h10-heart-rate-sensor/.

[22] Hettiarachchi I.T., Hanoun S., Nahavandi D., Nahavandi S. (2019). Validation of Polar OH1 optical heart rate sensor for moderate and high intensity physical activities. PLoS ONE.

[23] Bartels R., Neumamm L., Peçanha T., Carvalho A.R.S. (2017). SinusCor: An advanced tool for heart rate variability analysis. Biomed. Eng. Online.

[24] Smith A.L., Owen H., Reynolds K.J. (2013). Heart rate variability indices for very short-term (30 beat) analysis. Part 1: Survey and toolbox. J. Clin. Monit. Comput..

[25] Taelman J., Vandeput S., Spaepen A., Van Huffel S. (2009). Influence of mental stress on heart rate and heart rate variability. Proceedings of the 4th European Conference of the International Federation for Medical and Biological Engineering.

[26] Camm A.J., Malik M., Bigger J.T., Breithardt G., Cerutti S., Cohen R.J., Coumel P., Fallen E.L., Kennedy H.L., Kleiger R. (1996). Heart rate variability. Standards of measurement, physiological interpretation, and clinical use. Circulation.

[27] Hill S.G., Iavecchia H.P., Byers J.C., Bittner A.C., Zaklade A.L., Christ R.E. (1992). Comparison of four subjective workload rating scales. Hum. Factors.

[28] Marwan N., Romano M.C., Thiel M., Kurths J. (2007). Recurrence plots for the analysis of complex systems. Phys. Rep..

[29] Wallot S., Roepstorff A., Mønster D. (2016). Multidimensional Recurrence Quantification Analysis (MdRQA) for the analysis of multidimensional time-series: A software implementation in MATLAB and its application to group-level data in joint action. Front. Psychol..

[30] Donner R.V., Small M., Donges J.F., Marwan N., Zou Y., Xiang R., Kurths J. (2011). Recurrence-based time series analysis by means of complex network methods. Int. J. Bifurc. Chaos.

[31] UR Data (2011). Nonlinear Dynamical Systems Analysis for the Behavioral Sciences Using Real Data.

[32] Wallot S., Mønster D. (2018). Calculation of average mutual information (AMI) and false-nearest neighbors (FNN) for the estimation of embedding parameters of multidimensional time series in matlab. Front. Psychol..

[33] Raaijmakers S.F., Baars M., Schaap L., Paas F., Van Gog T. (2017). Effects of performance feedback valence on perceptions of invested mental effort. Learn. Instr..

[34] Stickel C., Ebner M., Steinbach-Nordmann S., Searle G., Holzinger A. (2009). Emotion detection: Application of the valence arousal space for rapid biological usability testing to enhance universal access. Proceedings of the Universal Access in Human-Computer Interaction. Addressing Diversity: 5th International Conference, UAHCI 2009, Held as Part of HCI International 2009.

[35] Wallot S., Mitkidis P., McGraw J.J., Roepstorff A. (2016). Beyond synchrony: Joint action in a complex production task reveals beneficial effects of decreased interpersonal synchrony. PLoS ONE.

